# Adult Neural Stem Cell Regulation by Small Non-coding RNAs: Physiological Significance and Pathological Implications

**DOI:** 10.3389/fncel.2021.781434

**Published:** 2022-01-04

**Authors:** Amber Penning, Giorgia Tosoni, Oihane Abiega, Pascal Bielefeld, Caterina Gasperini, Davide De Pietri Tonelli, Carlos P. Fitzsimons, Evgenia Salta

**Affiliations:** ^1^Laboratory of Neurogenesis and Neurodegeneration, Netherlands Institute for Neuroscience, Amsterdam, Netherlands; ^2^Swammerdam Institute for Life Sciences, Faculty of Science, University of Amsterdam, Amsterdam, Netherlands; ^3^Neurobiology of miRNAs Lab, Istituto Italiano di Tecnologia, Genova, Italy

**Keywords:** adult hippocampal neurogenesis, neurodegeneration, neural stem cells, small non-coding RNA, microRNA, piRNA

## Abstract

The adult neurogenic niches are complex multicellular systems, receiving regulatory input from a multitude of intracellular, juxtacrine, and paracrine signals and biological pathways. Within the niches, adult neural stem cells (aNSCs) generate astrocytic and neuronal progeny, with the latter predominating in physiological conditions. The new neurons generated from this neurogenic process are functionally linked to memory, cognition, and mood regulation, while much less is known about the functional contribution of aNSC-derived newborn astrocytes and adult-born oligodendrocytes. Accumulating evidence suggests that the deregulation of aNSCs and their progeny can impact, or can be impacted by, aging and several brain pathologies, including neurodevelopmental and mood disorders, neurodegenerative diseases, and also by insults, such as epileptic seizures, stroke, or traumatic brain injury. Hence, understanding the regulatory underpinnings of aNSC activation, differentiation, and fate commitment could help identify novel therapeutic avenues for a series of pathological conditions. Over the last two decades, small non-coding RNAs (sncRNAs) have emerged as key regulators of NSC fate determination in the adult neurogenic niches. In this review, we synthesize prior knowledge on how sncRNAs, such as microRNAs (miRNAs) and piwi-interacting RNAs (piRNAs), may impact NSC fate determination in the adult brain and we critically assess the functional significance of these events. We discuss the concepts that emerge from these examples and how they could be used to provide a framework for considering aNSC (de)regulation in the pathogenesis and treatment of neurological diseases.

## Introduction

After birth, most of the mammalian brain presents a cellular environment that is refractory to the generation of new neurons under physiological conditions (Kempermann, 2015). However, adult neural stem cells (aNSCs), residing within spatially restricted microenvironments of the adult brain, referred to as “niches” (Schofield, 1978; Lim et al., 2007), maintain the capability to self-renew, proliferate and generate differentiated progeny, mainly neurons, and, infrequently, astrocytes (Bottes et al., 2021). The subventricular zone (SVZ) of the lateral ventricles and the subgranular zone (SGZ) of the dentate gyrus (DG) in the hippocampus (Gage, 2019) are two of the main neurogenic niches in the adult mammalian brain. Here, we will focus on the adult hippocampal niche, because of the functional relevance of the hippocampus to memory and cognition, its link to neurodevelopmental and mood disorders and neurodegenerative diseases, and its sensitivity to insults such as epileptic seizures, stroke, or traumatic brain injury (for systematic reviews on the functional significance of the adult hippocampal neurogenic niche, see Kempermann et al., 2018; Duque and Spector, 2019; Snyder, 2019; Lucassen et al., 2020; Babcock et al., 2021; Denoth-Lippuner and Jessberger, 2021).

**Figure 1:**
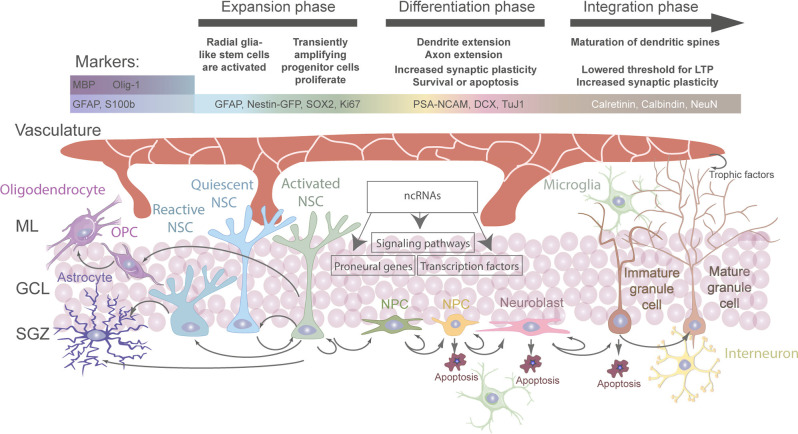
Regulation and fate of aNSC in the adult hippocampal stem cell niche. Overview of all cell types involved directly or indirectly in the regulation of stem cell activation and fate determination in the adult hippocampus. Cell-intrinsic and extrinsic signals regulate the different stages of aNSC quiescence, activation, neurogenesis, astrogliogenesis, and oligodendrogliogenesis in the adult dentate gyrus (DG).

Generally, stem cell niches are characterized by the ability to anatomically host stem cells and functionally control their development *in vivo* in order to adapt to organismal and environmental requirements (Alonso, 2009; Li and Guo, 2021). At the adult hippocampal neurogenic niche, this is achieved by a complex crosstalk between aNSCs and other cell types present (Figure 1; Vicidomini et al., 2020). More specifically, the milieu of the adult hippocampal neurogenic niche is tightly regulated by intracellular and extracellular signals (e.g. aging, stress, physical exercise, environmental enrichment, learning, pathological alterations, neurotrophins, growth factors, signaling pathways, extracellular matrix mediators and extracellular vesicles, vasculature-secreted factors and local circuit activitiy; for systematic reviews, see Balu and Lucki, 2009; Aimone et al., 2014; Baptista and Andrade, 2018; Bonafina et al., 2020; Losurdo and Grilli, 2020; Vicidomini et al., 2020). Thereby, a regulated balance between aNSC quiescence, proliferation, self-renewal, differentiation, and fate choice contributes to lifelong hippocampal plasticity and fitness, while preventing neurodegeneration and other pathologies (Faigle and Song, 2013; Fitzsimons et al., 2014; Yao et al., 2016; Lopatina et al., 2019; Toda et al., 2019; Vicidomini et al., 2020; Kim et al., 2021; Li and Guo, 2021). Hence, understanding the regulation of aNSCs and their crosstalk with other cell types within the hippocampal neurogenic niche is indispensable to fully map the mechanisms governing their fate determination under physiological conditions and in the diseased brain.

A considerable part of the regulatory complexity required for proper aNSC homeostasis in the adult hippocampal niche is executed by non-protein-coding RNAs (ncRNAs), which comprise a significant proportion of the transcriptional output of the eukaryotic genome, contributing to both housekeeping and key regulatory processes (Carninci et al., 2005; Dunham et al., 2012; Salta and De Strooper, 2012, 2017b; Salvatori et al., 2020). These functions are regarded as particularly relevant for cells such as aNSCs, which require fast adaptation of transcriptome and protein abundance, especially when transitioning through cellular states while undergoing fate decisions (Encinas and Fitzsimons, 2017). However, given that the majority of ncRNA species are still functionally uncharacterized, it remains unclear how the diverse ncRNA-mediated pathways regulate fate determination in aNSCs. A deeper understanding of how ncRNA functions impact aNSCs may provide a better mechanistic appreciation of physiological brain development, its plasticity, and the disorders arising from the deregulation of aNSCs. Such insights may also prove pivotal for the next generation of RNA-based therapeutics targeting abnormal gene expression in aNSCs, in order to prevent, counteract or correct disruption of their homeostasis, activation and differentiation.

Here, we review recent discoveries reporting the regulation of cell intrinsic programs by small ncRNAs (sncRNAs) in several immature cell types of the adult hippocampal neurogenic niche, we discuss the mechanisms by which they impact fate determination of these cell populations and how, sometimes, sncRNAs may interact with each other to act as intercellular signals and regulate neighboring cells within the hippocampal neurogenic niche.

## Adult Neural Stem Cells in The Hippocampus: Complexity and Regulation

In the adult hippocampus, aNSCs occupy a narrow area between the hilus and the granular cell layer of the DG, known as SGZ (Kempermann et al., 2004), which forms part of the neurogenic niche (Figure 1). ANSCs and their immature progeny in the adult hippocampal neurogenic niche have often been classified using divergent nomenclature, which we summarize in Table 1. ANSCs are also known as “radial glia-like cells” (RGLs), due to their similarity to embryonic radial glial NSCs, and “quiescent neural stem cells” or “type 1 cells” (Table 1; Kempermann et al., 2004; Encinas et al., 2011). ANSCs are maintained in a reversible cell cycle arrest state, termed quiescence. Daughter cells generated by initial mitotic divisions of activated aNSCs exhibit reduced self-renewal and increased lineage restriction and proliferative potential (Berg et al., 2018). These highly proliferative progeny are functionally classified as “(transiently) amplifying (neural) progenitors” (herein collectively termed neural progenitor cells, “NPCs”), “intermediate progenitors”, or based on marker expression, as “type 2a cells” with a glial phenotype, and “type 2b cells” with a neuronal phenotype (Table 1). NPCs generate immature neuroblasts, also termed “type 3 cells,” which exit the cell cycle to become immature neurons first and, over a period of several weeks, newborn mature dentate granule neurons (“granule cells”, DGCs; Table 1; Encinas et al., 2011; Kempermann, 2015; Berg et al., 2018). Oligodendrocyte precursor cells (OPCs) are also present in the adult DG, yet to a significantly lesser extent, and not found to be restricted to the subgranular zone (Encinas et al., 2011). In addition, neuron-glial antigen 2 (NG2, a chondroitin sulfate proteoglycan)-expressing progenitor cells are present and continue to proliferate in the adult CNS (Ffrench-Constant and Raff, 1986; Wolswijk and Noble, 1989), and may represent a distinct class of proliferating glial cells in the adult hippocampus (Klempin et al., 2011; Schouten et al., 2020). Yet, their intrinsic stem cell potential and functional contribution are not well-characterized, with some studies reporting that NG2-expressing cells are multipotent progenitors that generate astrocytes and mature neurons in the postnatal hippocampus (Belachew et al., 2003), while others suggest that they may be OPCs (Dawson et al., 2000). Of note, single-cell transcriptomic profiling studies in the mouse DG have demonstrated that cells of the neurogenic lineage may co-exist in a heterogeneous continuum of cell states (Shin et al., 2015; Artegiani et al., 2017). A similar heterogeneity has been reported for oligodendrocytic and astrocytic precursors in several brain regions, including, among others, cortical, subcortical, and hippocampal subfields (Steindler and Laywell, 2003; Marques et al., 2016).

**Table 1:** Terminology used to describe aNSCs and their progeny.

Terminology	Others	Cellular state	Reference(s)Our review
Adult neural stem cells (aNSCs)	Radial glia-like cells Quiescent neural stem cells Type-1 cells	Quiescent/Activated/Proliferative	Kempermann et al. (2004) and Encinas et al. (2011)
Neural progenitor cells (NPCs)	Intermediate progenitors Transiently amplifying neural progenitors Type 2a cells (glial phenotype) Type 2b cells (neuronal phenotype)	Proliferative	Kempermann et al. (2004), Encinas et al. (2011), and Berg et al. (2018)
Oligodendrocyte precursor cells	NG2-positive cells	Proliferative	Dawson et al. (2000) and Belachew et al. (2003)
Neuroblasts	Type 3 cells	Largely postmitotic	Kempermann et al. (2004), Encinas et al. (2011), and Berg et al. (2018)

Besides aNSCs and their progeny, the adult hippocampal niche is a dynamic structure containing multiple other cell types (Figure 1). The functional intercellular crosstalk among the niche-resident cells allows for proper maintenance of the aNSC pool, progenitor proliferation, differentiation, survival, and maturation (Vicidomini et al., 2020). Of note, aNSCs and NPCs can regulate their own homeostasis, as well as the differentiation and maturation of their progeny, through autocrine and paracrine signals (Schultheiß et al., 2013; Vicidomini et al., 2020; Shariq et al., 2021). Similarly, hippocampal niche-resident astrocytes have been shown to provide both structural and functional support to aNSCs and their progeny, and to control cell proliferation, differentiation, migration, and synaptic integration of adult-born granule neurons (Bonafina et al., 2020). Moreover, microglia, the brain-resident immune cells, actively regulate multiple phases of neurogenesis (Butovsky et al., 2006; Ekdahl et al., 2009; Woodbury et al., 2015; Pérez-Rodríguez et al., 2021). In addition, blood vessels localize within the neurogenic niche and their capillary loops are found in close proximity to aNSCs and dividing NPCs (Licht and Keshet, 2015). As such, the vascular compartment can impact neurogenesis through direct contact with, and paracrine signaling from, endothelial and mural cells that make up blood vessels, or indirectly, *via* transporting systemic signals (in the form of soluble factors) from blood circulation into the neurogenic niche (Fitzsimons et al., 2013; Lee et al., 2013; Villeda et al., 2014; Licht and Keshet, 2015; Smith et al., 2015; Bátiz et al., 2016; Schouten et al., 2020). Last, mature hippocampal DGCs can modulate aNSC homeostasis *via* cell autonomously secreted factors and by recruiting circuit mechanisms involving, among others, local inhibitory interneurons and mossy cells (Gobeske et al., 2009; Ma et al., 2009; Jang et al., 2013; Brooker et al., 2017).

How this melting pot of cells and signals is orchestrated to eventually direct aNSCs and other niche-resident progenitors towards quiescence, neurogenesis, astrogliogenesis, and oligodendrogliogenesis is of particular importance for understanding the functional relevance of these processes in both the healthy and the diseased adult brain. In the next sections, we will focus on the pivotal regulatory role of sncRNAs as cell-intrinsic or -extrinsic signals and will critically discuss the emerging evidence supporting distinct key functions for these small but mighty regulators in the adult hippocampal niche.

## Small Non-Coding RNAs

**Figure 2:**
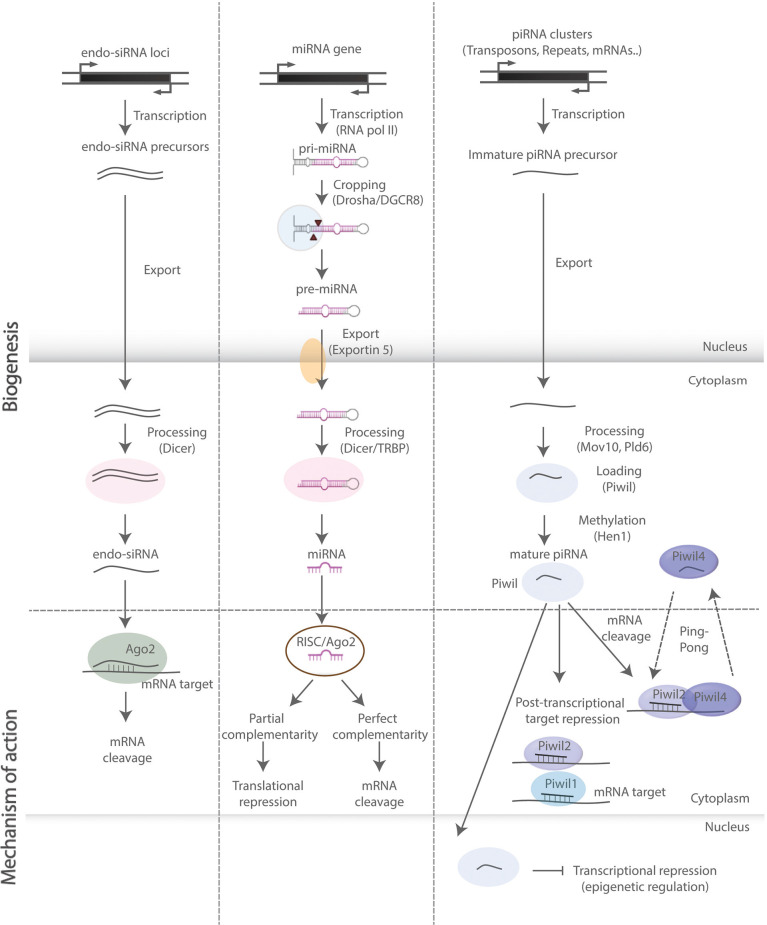
SncRNA biogenesis and mode of action. Schematic representation of biosynthetic and regulatory pathways of the sncRNA species discussed in this review. Endo-siRNAs are short double-stranded RNAs, processed by Dicer and incorporated into the RNA-induced ribonucleoprotein silencing complex (RISC), upon which they bind onto target mRNAs and induce mRNA cleavage. MiRNAs are primarily transcribed by RNA polymerase II and subsequently processed by—among others—Drosha and Dicer into double-stranded miRNA precursor molecules. The guide strand is then incorporated into RISC leading to translational repression and mRNA degradation upon base pairing with complementary sites on mRNA targets. PiRNAs are processed in a Dicer-independent manner and act through binding with PIWI-clade proteins exerting both transcriptional and post-transcriptional regulatory effects (“ping-pong” amplification loop).

NcRNAs are RNA molecules not translated into proteins. They are traditionally classified based on their average size into small (<200 nucleotides) and long (>200 nucleotides) species (Hombach and Kretz, 2016; Salvatori et al., 2020). These molecules are highly versatile and exhibit a very diverse mechanistic repertoire, ranging from regulation of chromatin state, transcription, epigenetic events, alternative splicing, and RNA interference (RNAi)-mediated gene silencing, to effects on mRNA stability, translational regulation, and sequestration of RNA and proteins from their natural targets in the intracellular space (Schouten et al., 2012; Salta and De Strooper, 2017b; Figure 2). In this review, we focus on regulatory sncRNAs (Figure 2), and in particular on microRNAs (miRNAs), which are the most studied sncRNA subclass with well-documented and emerging roles in the regulation of aNSCs and their progeny. We also discuss the functions of PIWI-interacting RNAs (piRNAs), which are rapidly emerging as important regulators of somatic stem cells. For further information on long ncRNAs in the central nervous system (CNS), readers are invited to consider several previous reviews that cover the topic in more detail (Salta and De Strooper, 2017b; Zhang et al., 2019b).

MiRNAs are a class of small (19–22 nucleotides in length) single-stranded ncRNAs. AGO proteins associate with miRNAs and other sncRNAs acting in RNA-based silencing mechanisms by inhibiting protein synthesis and altering RNA stability (Hutvagner and Simard, 2008; Figure 2). Their functions are particularly important for the fine-tuning of gene expression in brain development, physiology, and homeostasis (Saba and Schratt, 2010; Barca-Mayo and De Pietri Tonelli, 2014; Zhang et al., 2019b). Of note, miRNAs may repress individual targets only to a limited extent (Baek et al., 2008; Selbach et al., 2008). Thereby, their regulatory potential may often rely on the synergistic action of several miRNAs over distinct sets of transcripts, which in aNSCs often encode proteins that share similar or converging biological functions (Barca-Mayo and De Pietri Tonelli, 2014; Schouten et al., 2015; Pons-Espinal et al., 2017).

PiRNAs are a class of animal-specific single-stranded sncRNAs defined by their ability to associate with PIWI proteins, a subclass of Dicer-binding AGO proteins (Doyle et al., 2012). PiRNAs are longer (21–35 nucleotides in length) than miRNAs and endogenous siRNAs, and unlike the former classes, they are processed in a Dicer-independent manner, bear 2′-O-methyl-modified 3′ termini and only function through binding with PIWI-clade proteins, rather than AGO-clade proteins (Figure 2). Although much less studied than miRNAs, piRNAs are present in the mammalian CNS, while nearly 30,000 neuronal piRNAs have been identified in the mouse brain throughout development, suggesting that they are expressed and dynamically regulated during brain development (Ghoshes et al., 2016). In addition, several studies have shown that piRNAs might regulate neuronal function and synaptic plasticity in the mammalian brain (Wakisaka and Imai, 2019).

**Figure 3:**
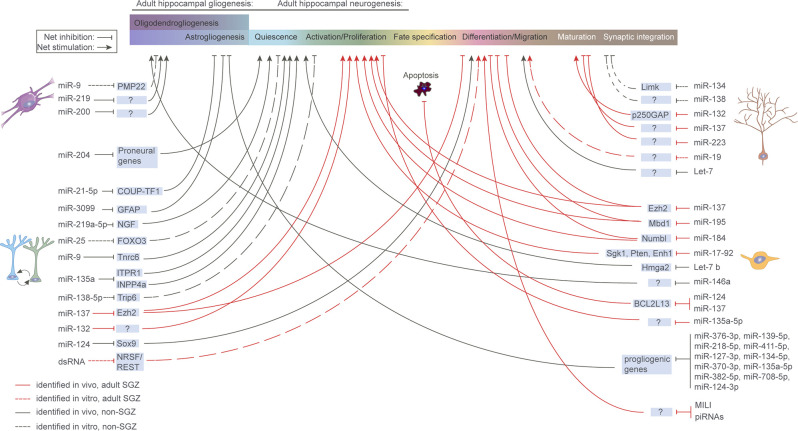
microRNA regulation of aNSCs and their progeny. Overview of miRNAs known to be functionally implicated in aNSC quiescence, activation, fate determination, and progeny maturation under physiological conditions in the murine hippocampus. Continuous red line: *in vivo* study, refers to adult SGZ; Dashed red line: *in vitro* study, refers to adult SGZ; Continuous black line: *in vivo* study, refers to systems other than SGZ; Dashed black line: *in vitro* study, refers to systems other than SGZ.

The disruption of miRNA and piRNA expression has been linked to a multitude of brain pathologies, including neurodegenerative diseases (Salta and De Strooper, 2012, 2017b; Wakisaka and Imai, 2019; Wu and Kuo, 2020; Huang and Wong, 2021), psychiatric (Yoshino and Dwivedi, 2020) and neurodevelopmental disorders (Barca-Mayo and De Pietri Tonelli, 2014; Zhang et al., 2019d). In agreement with their general involvement in health and disease, accumulating evidence links sncRNAs to aNSC regulation under both physiological (Figure 3) and pathological conditions (Table 2). In the following sections, we discuss the existing evidence demonstrating how sncRNA species, particularly miRNAs and piRNAs, can impact aNSC maintenance, fate determination, differentiation, and maturation of their progeny.

**Table 2:** MicroRNAs implicated in the regulation of aNSCs and their progeny in neurological diseases.

Neurogenesis			
Non-coding RNA(s)	Disease	Functional relevance	References
miR-132	Alzheimer’s disease	-Decreased expression in AD hippocampus. -Exerts proneurogenic effects in aNSCs and their progeny.	Walgrave et al. (2021a)
miR-124	Parkinson’s disease	Increases the number of neuroblasts reaching the granular cell layer of the olfactory bulb (SVZ).	Saraiva et al. (2016)
miR-30 family	Depression	- Decreased expression in the mouse model of depression. - Mediates chronic stress-induced depression-like phenotype by altering hippocampal neurogenesis and neuroplasticity *via* controlling epigenetic and transcription regulators, such as Mll3 and Runx1; and cell signaling regulators, like Socs3, Ppp3r1, Gpr125, and Nrp1.	Khandelwal et al. (2019)
miR-19	Schizophrenia	Upregulated in SGZ-NPCs.	Han et al. (2016)
miR-124 and miR-137	Epilepsy	Concomitant knockdown of the two prevents hippocampal aNSC loss upon non-convulsive seizures.	Bielefeld et al. (2017) and Bielefeld et al. (2019)
Astrogliogenesis/Reactive astrogliosis			
Non-coding RNA(s)	Disease	Functional relevance	References
miR-132	Epilepsy	- Increased expression in reactive astrocytes in epileptogenic human and rat hippocampus. - Reduces pro-epileptogenic factors in cultured astrocytes.	Korotkov et al. (2020)
miR-146a	Epilepsy	- Increased expression in reactive astrocytes in epileptogenic human and rat hippocampus.	Aronica et al. (2010)
	Down syndrome	- Increased expression in reactive astrocytes in the brain of individuals with Down syndrome.	Arena et al. (2017)
miR-181a	Ischemia	Its inhibition induces neurogenesis partly *via* astrocytic dedifferentiation in the hippocampus.	Griffiths et al. (2019)
miR-302/367	Alzheimer’s disease	Induce conversion of reactive astrocytes to neurons in DG.	Ghasemi-Kasman et al. (2018)
Oligodendrogliogenesis			
Non-coding RNA(s)	Disease	Functional relevance	References
miR-146a	Stroke	Induces remyelination by increasing differentiation of OPCs to oligodendrocytes.	Santra et al. (2014) and Liu et al. (2017)
	Multiple sclerosis	Enhances myelination and increased OPC differentiation.	Zhang et al. (2019a)
miR-23a, miR-219a, and miR-338	Demyelinating insult	Promote oligodendrocytic differentiation and remyelination.	Santos et al. (2020)

## microRNAs

### Regulation of aNSC Quiescence

#### Effects in Physiological Conditions

aNSCs in the adult brain are typically quiescent (Doetsch et al., 1999), allowing for reactivation and cell cycle re-entry to generate neuronal and gliogenic progeny upon distinct environmental stimuli (Delgado et al., 2021). Maintaining the quiescent state of aNSCs is thought to be critical for preserving the NSC pool during aging (Encinas et al., 2011), as overactivation of aNSCs may deplete the aNSC pool (Sierra et al., 2015). Thus, this intricate balance between aNSC quiescence and activation drives neuro- and gliogenesis, while safeguarding a resident aNSC population from decay (Harris et al., 2021). Over the recent years, extensive studies have highlighted several cell-intrinsic and extrinsic cues, as well as systemic factors, that play pivotal roles in the regulation of aNSC quiescence, both in health and disease (Figure 1).

Key signaling pathways acting as regulators of NSC quiescence include, among others, Notch (Ehm et al., 2010; Imayoshi et al., 2010; Lugert et al., 2010), ephrins in endothelial cells (Ottone et al., 2014), cadherins in ependymal cells (Porlan et al., 2014), BMP, WNT (Li and Clevers, 2010; Mira et al., 2010), GABAergic (Song et al., 2012; Giachino et al., 2014), and glutamatergic signaling (Lugert et al., 2010; Sierra et al., 2015; Bielefeld et al., 2019). For an extensive overview of all signaling pathways and proteins involved in maintaining aNSC quiescence, we refer to an excellent recent review (Urbán et al., 2019).

Although all these molecular cascades provide potential targets for ncRNA control, only a few sncRNAs have been identified that regulate aNSC quiescence, all of them being miRNAs (Figure 3). A key miRNA controlling NSC quiescence is miR-9. As will be discussed later, miR-9 is classically known to be a regulator of the NPC proliferation/differentiation balance when localized to the cytoplasm through Tlx, Foxo1 and Notch (Zhao et al., 2009; Kim et al., 2015). However, subsets of aNSCs are extensively enriched for miR-9. When localized to the nucleus, miR-9 acts as a rheostat for aNSC activation/quiescence, through its target TNRC6 and the Notch pathway (Katz et al., 2016).

MiR-204 has been shown to be transferred from the choroid plexus to the cerebrospinal fluid, allowing long-distance regulatory control of aNSCs residing in the SVZ. Silencing miR-204 results in significant activation of quiescent aNSCs, through the upregulation of a plethora of neurogenic target genes (Lepko et al., 2019).

Another example of systemic regulation of aNSC quiescence under the control of sncRNAs is Foxo3, a downstream factor of the IGF signaling pathway. Foxo3 is required to maintain NSC quiescence in both the SVZ and SGZ (Paik et al., 2009; Renault et al., 2009), and forms a feedback loop with the miR-106b-25 cluster, which is encoded in an intronic region of a Foxo3 target gene (Mcm7; Brett et al., 2011). The Foxo3 mRNA is one of the predicted targets of the transcribed miR-25, creating a negative feedback loop controlling aNSC quiescence. Yet, the involvement of this feedback loop in the regulation of NSC quiescence has not been experimentally validated *in vivo*.

#### Effects in Pathological Conditions

ANSC activation is linked to several neurological pathologies, of which epileptic seizures have been widely validated in animal models (Lugert et al., 2010; Sierra et al., 2015). Overactivation of aNSCs under pathological conditions results in a rapid depletion of the aNSC pool and a concomitant decrease in the neurogenic capacity in the DG (Sierra et al., 2015), suggesting an association with the cognitive deficits (Cho et al., 2015). Several studies have mapped the ncRNA response to seizures in both mice and humans (Schouten et al., 2015; Bencurova et al., 2017; Bielefeld et al., 2017; Brennan and Henshall, 2020). Interestingly, the expression of both the miR-106b-25 cluster and miR-9 in the hippocampus are altered at different stages after seizure onset (McKiernan et al., 2012; Risbud and Porter, 2013; Wang et al., 2015). However, none of these alterations or their potential role in the regulation of NSC activation have been experimentally validated. Of note, a lack of aNSC activation is thought to be involved in neuropsychiatric disorders, of which depression is the most extensively studied. As antidepressant drugs increase neurogenesis, and electroconvulsive shock therapy induces aNSC activation, it has been suggested that a lack of aNSC activation may underlie the development of depression (Encinas et al., 2006; Hanson et al., 2011). However, clear evidence for a potential role of sncRNAs in this imbalance of aNSC activation has not been reported yet.

### Regulation of Neurogenesis From aNSCs

A first demonstration of the dependency of NSCs on miRNA regulation comes from studies in which one of the essential miRNA-processing enzymes, Dicer, was deleted in adult or embryonic NSCs, precluding global miRNA generation (Saurat et al., 2013; Pons-Espinal et al., 2017; Xu et al., 2020). ANSC-specific ablation of Dicer in mouse DG and rescue with mature miRNAs confirmed that miRNAs, but not miRNA-independent Dicer functions, are required for the generation of adult-born DGCs (Pons-Espinal et al., 2017). However, in the absence of reliable cell type-specific markers for the selective genetic targeting of aNSCs, differentiating between NSC-specific and non-specific effects remains challenging. Another interesting observation is that miRNAs respond to some of the extrinsic stimuli that act as triggers of AHN in DG. More specifically, miRNAs were shown to mediate some of the enhancing effects of both environmental enrichment and physical exercise on AHN (Barak et al., 2013; Pons-Espinal et al., 2019; Walgrave et al., 2021a), possibly placing miRNAs upstream of signaling pathways and molecular regulators that induce AHN. In addition, miRNAs frequently function in concerted action with transcription factors and chromatin modifiers, as we discuss in more detail in the following sections. Thereby, miRNAs provide an additional layer to ensure the robustness of gene regulatory programs dictating the fate transitions along the neurogenic trajectory (Encinas and Fitzsimons, 2017; Stappert et al., 2018).

#### Effects in Physiological Conditions: Early Neurogenic Events

Besides evidence derived from global inhibition of miRNA biogenesis, individual miRNAs, or miRNA networks can also induce either widespread effects on the adult neurogenic process or preferentially regulate distinct stages along the neurogenic trajectory from aNSCs to adult-born neurons. The miRNA impact on the net outcome of neuronal differentiation can be attributed to the regulation of upstream processes, direct targeting of differentiation effectors, or both. However, non-cell-autonomous effects mediated by other niche-resident cells cannot be ruled out, given the lack of aNSC/NPC targeting specificity in the transgenic mice generation strategy. Lentiviral knockdown of miR-132, primarily in aNSCs in the mouse DG, blocked the running-induced increase in NPC proliferation and neuronal differentiation, suggesting that miR-132 is required for the running-mediated effects on AHN (Walgrave et al., 2021a). Although systematic validation of the pertinent miR-132 targetome in aNSCs is still pending, miR-132 overexpression induced broad effects in genes involved in cell cycle regulation and neuronal differentiation, as assessed by single-cell RNA-sequencing in Nestin-positive niche-resident cells. Functioning as a gatekeeper of the balance between proliferation and differentiation, miR-138-5p acts through inactivation of the TRIP6, a molecule that is necessary and sufficient to maintain aNSC proliferation and self-renewal capacity. Downregulation of miR-138-5p *in vitro* promotes aNSC proliferation and inhibits neuronal differentiation, whereas miR-138-5p overexpression exerts opposite effects (Wang et al., 2017b). While such shifts are easier to monitor in *in vitro* aNSC mono-cultures, differentiating between effects on distinct stages of aNSC self-renewal and NPC proliferation *in vivo* would require further systematic fate mapping.

Interestingly, miR-137, a miRNA epigenetically repressed by MeCP2, a DNA methyl-CpG-binding protein also regulating AHN, was shown to induce NPC proliferation in SGZ at the expense of neuronal differentiation, upon retroviral overexpression in the adult DG (Szulwach et al., 2010). In a feedback regulatory loop back onto chromatin, Ezh2, a histone methyltransferase and polycomb group protein, was identified as a direct target of miR-137, mediating its effects on AHN and exemplifying the complex miRNA interactomes in the adult hippocampal neurogenic niche. A similar interplay between miRNA and epigenetic regulation was reported for miR-184 and miR-195, which regulate the balance between aNSC activation/proliferation and differentiation in the adult SGZ niche by forming a regulatory network with their epigenetic repressor, Mbd1. MiR-184 then targets Numbl, an important player during brain development (Liu et al., 2010), whereas miR-195 signals back to Mbd1 (Liu et al., 2013). In the same vein, let-7b regulates the expression of Hmga2, a small chromatin-associated protein that can modulate transcription by altering chromatin structure (Nishini et al., 2008). Aging-associated Hmga2 deficiency in adult mouse SVZ, which is partially mediated by increased let-7b expression, reduces the self-renewal potential of aNSC in the aged brain. Apart from a pivotal role as a rheostat between aNSC quiescence and activation (Katz et al., 2016), as discussed earlier, miR-9 was also reported to suppress aNSC proliferation and accelerate neuronal differentiation in the embryonic mouse SVZ, *via* a negative feedback regulatory loop with its target, TLX (Zhao et al., 2009). These observations were confirmed in primary NSC/NPC cultures derived from neonatal mouse brain, where miR-9 was shown to positively regulate neuronal differentiation *via* a regulatory cascade involving Foxo1 and Notch signaling (Kim et al., 2015). Although suggestive of a potent miR-9-dependent gene regulatory network fine-tuning NPC proliferation and neuronal differentiation during embryonic and early postnatal stages, a similar role for miR-9 in the adult brain has not yet been reported. Another miRNA with crucial functions in mammalian CNS is miR-124, the most abundant miRNA in the adult brain (Lagos-Quintana et al., 2002). MiR-124 was reported to be a key determinant of neuronal fate in aNSCs in mouse SVZ (Åkerblom et al., 2012), where miR-124 overexpression induces neuronal differentiation by directly targeting Sox9, a key transcription factor for adult neurogenesis (Cheng et al., 2009). Of note, miR-124 also regulates and is regulated by REST, a primary transcriptional repressor responsible for inhibiting the expression of neuronal genes in non-neuronal cells (Conaco et al., 2006). Whether the miR-124/REST regulatory loop is also involved in AHN remains to be assessed.

#### Effects in Physiological Conditions: Late Neurogenic Events

Newly born immature neurons in the SVZ and SGZ need to migrate over distinct distances that depend on the neurogenic niche in which they developed. Let-7 and miR-19 regulate the migration of adult-born neurons in the olfactory bulb and the DG, respectively (Han et al., 2016; Petri et al., 2017). The regulation of the maturation of newborn neurons at late neurogenic stages has also been documented for several miRNAs. As most of these studies employed either *in vitro* or embryonic systems, it is possible that these regulatory mechanisms are not applicable in the adult brain, and even if they were, they may represent generalized miRNA functions, and not adult-born neuron-specific phenomena. These limitations further complicate conclusions regarding AHN-specific miRNA-dependent effects on memory formation and cognition (for a review on miRNA roles in memory formation, see Saab and Mansuy, 2014). Along these lines, an additional putative role for miR-124 in the neurogenic process is the induction of both dendritic arborization and axonal growth *via* repression of RhoG, a small GTPase involved in neuritogenesis (Franke et al., 2012). However, these effects have only been monitored in cultured hippocampal neurons. Reversely, miR-134 and miR-138, a brain-specific and a brain-enriched miRNA, respectively, are localized to the synapto-dendritic compartment and were shown to negatively regulate the development of dendritic spines in hippocampal neurons *in vitro* (Schratt et al., 2006; Siegel et al., 2009). Interestingly, miR-134 acts by targeting Limk1 in response to neuronal stimulation by BDNF, a key modulator of neuronal plasticity (Schratt et al., 2006). Another neuronal activity- and CREB-regulated miRNA, miR-132, was also previously shown to promote neurite outgrowth, both in cultured cortical neurons *via* its direct target, p250GAP (Vo et al., 2005), and *in vivo* (Magill et al., 2010; Luikart et al., 2011; Pathania et al., 2012; Walgrave et al., 2021a). Of note, miR-132 is emerging as a key component of structural plasticity networks regulating dendritic arborization, survival, and synaptic integration, both in postnatal neurogenesis at the SVZ (Pathania et al., 2012) and cell-autonomously, in adult-born neurons in DG (Magill et al., 2010; Luikart et al., 2011; Walgrave et al., 2021a). Conversely, both miR-137 and miR-223 negatively impact dendritic outgrowth in newly born GCs in the adult mouse brain upon retroviral targeting of adult NPCs (Smrt et al., 2010; Harraz et al., 2014). Taken together, these findings strongly support a multilayered role for miRNAs in the neurogenic process in general, and in AHN in particular. Considering the contextualization component of many miRNA regulatory effects, validation studies in the (aging) brain are required to assess the conservation of similar miRNA-mediated mechanisms.

#### Effects in Pathological Conditions

AHN has been shown to be impaired by several pathologies, including neurodegenerative diseases, mood disorders, epilepsy, and substance use disorders. Given the pivotal role of miRNAs in controlling critical steps of the neurogenic process discussed before, the link between miRNA deregulation and these pathologies in the context of neurogenesis has been investigated on several occasions, although direct functional links have mainly been drawn from *in vitro* studies and rodent models.

##### Neurodegenerative Disorders

Alterations in adult neurogenesis are a common phenomenon in several neurodegenerative disorders, which could potentially contribute to their pathophysiology. For example, aberrant AHN has been reported in Alzheimer’s disease (AD) patients and mouse models (Babcock et al., 2021), and recent findings suggest that in AD patients, the number and the maturation of newly born GCs progressively decline as AD pathology advances (Moreno-Jiménez et al., 2019; Tobin et al., 2019). Restoring adult hippocampal neurogenesis deficits in several AD mouse models was shown to improve AHN-related memory (Choi et al., 2018; Kim et al., 2019; Walgrave et al., 2021a). Intriguingly, miR-132 is consistently downregulated in the hippocampus of human AD patients (Salta and De Strooper, 2017a) and has been recently shown to be a potent regulator of AHN, exerting cell-autonomous proneurogenic effects in aNSCs and their progeny. Furthermore, it was shown that miR-132 replacement in adult mouse AD hippocampus restores AHN and relevant memory deficits, opening up novel avenues for possible therapeutic approaches targeting miR-132 in neurodegeneration (Walgrave et al., 2021b).

Another neurodegenerative disease associated with impaired AHN is Parkinson’s disease (PD; reviewed in Lim et al., 2018). Signaling pathways implicated in the neuroinflammatory response and autophagy are crucial in PD pathogenesis and could potentially be orchestrated by different miRNAs, among other regulators (Nuzziello and Liguori, 2019). However, to date, the only evidence linking miRNAs, PD, and adult neurogenesis involves the SVZ, where neurogenesis is found markedly impaired in the PD brain. Assessing the neurogenic and migratory potential of SVZ-derived neuroblasts, both in physiological conditions and in a 6-OHDA-mouse model of PD, showed that single administration of nanoparticle-encapsulated miR-124, was able to promote migration of neuroblasts reaching the granular cell layer of the olfactory bulb and ameliorating motor symptoms (Saraiva et al., 2016).

##### Mood and Psychiatric Disorders

In addition to neurodegenerative diseases, mood and psychiatric disorders have also been linked to AHN. Aberrant AHN has been associated with depression, schizophrenia, and anxiety (Kang et al., 2016). Evidence shows that antidepressants increase AHN and that enhanced AHN is sufficient to promote resilience to anxiety and depression-related behaviors in a mouse model of stress (Hill et al., 2015; Kang et al., 2016; Planchez et al., 2021). Recent studies suggested once again a putative role for miRNAs in the interplay between AHN and mood disorders. The miR-30 family was shown to mediate a chronic stress-induced depression-like phenotype by altering hippocampal neurogenesis and neuroplasticity *via* controlling epigenetic, transcription, and cell signaling regulators (Khandelwal et al., 2019).

Abnormal migration of adult-born hippocampal neurons has been described in schizophrenia (Duan et al., 2007; Kim et al., 2009), and rare inherited copy number variants of RAPGEF2 have been associated with familiar schizophrenia (Xu et al., 2009). In a study investigating the role of miR-19 and RAPGEF2 in schizophrenia using patient-derived hippocampal NPCs (SZ-NPCs), miR-19 was found upregulated in SZ-NPCs, and conservation of the regulation of RAPGEF2 by miR-19 was demonstrated in human embryonic stem cell-derived hippocampal NPCs (Han et al., 2016). Of note, miRNA-19 belongs to the family of polycistronic miR-17 clusters, including also miR-17-92 and miR-106b-25b, which, as discussed above, have been shown to be important for aNSC proliferation (Brett et al., 2011; Jin et al., 2016). Hence, it would be interesting to assess whether the other members of the miR-17 clusters are also altered in schizophrenia and if they contribute to pathology.

##### Epilepsy

Temporal lobe epilepsy (TLE), characterized by seizures originating in many cases from the hippocampal region, is the most common form of epilepsy. AHN is particularly sensitive to seizures. Pioneering studies showed that epileptic seizures cause a significant increase in the short-term proliferation rate of NSCs, while diminishing long-term proliferation (Parent et al., 1997), and that newborn neurons in the epileptic brain also display several morphological and functional alterations, suggesting a possible role for aberrant AHN in epilepsy (Scharfman et al., 2000; Dashtipour et al., 2001; Murphy et al., 2011, 2012; Wood et al., 2011; Bielefeld et al., 2014). Over the recent years, it has been shown that many miRNAs known to regulate AHN are also deregulated during acute seizure events or chronic epilepsy, particularly in the DG (Schouten et al., 2015), providing further insights into the role of AHN in the context of epilepsy (Bielefeld et al., 2017). Of note, concomitant knockdown of miR-124 and miR-137 has been shown to prevent hippocampal aNSC loss upon non-convulsive seizures (Bielefeld et al., 2019).

### Regulation of Adult Astrogliogenesis

#### Effects in Physiological Conditions

New astrocytes are continuously generated at low levels alongside the new neurons in the adult hippocampus (Kuhn et al., 1996; Suh et al., 2007; Bottes et al., 2021). However, there is still not a clear consensus as to whether astrocytes are generated by multipotent NSCs or by a distinct type of stem cells, which have some characteristics of differentiated astrocytes (Alvarez-Buylla et al., 2001; Song et al., 2002; Steiner et al., 2004). On the molecular level, two transcriptional mechanisms controlling neuron-astroglia fate decisions in the adult hippocampus have been recently described, the Notch2-Id4 axis, possibly in synergy with transforming growth factor-β (TGF-β)/bone morphogenetic protein (BMP) pathway (Zhang et al., 2019c), and the COUP-TFI(Nr2f1) pathway (Bonzano et al., 2018).

Despite several studies indicating that miRNAs are essential for the developmental switch from neurogenesis to gliogenesis in mice (Zheng et al., 2010; Shenoy et al., 2015; Tsuyama et al., 2015), currently, understanding of their role in the control of adult astrogliogenesis is missing. As mentioned earlier, a study addressing this question showed that loss of Dicer-dependent miRNA impairs neurogenesis but not astrogliogenesis in the adult hippocampus *in vivo* (Pons-Espinal et al., 2017). However, different mechanisms could account for this observation. Firstly, it is possible that different subtypes of neural and glial progenitor cells exist in the adult hippocampal niche (Pilz et al., 2018; Bottes et al., 2021), which differentially respond to miRNA depletion. Secondly, astrocytes in glia-like hippocampal aNSCs might represent a “default” developmental path rather than a true fate choice (Encinas et al., 2011; Kempermann, 2011), and thus, their generation could be less dependent on miRNAs. Finally, it is also possible that miRNAs need to suppress glial fates in order to sustain neurogenesis. This hypothesis would be consistent with the evidence in mouse cortical development, where COUP-TF1 is actively suppressed by miR-21-5p (Terrigno et al., 2018) and GFAP by miR-3099 (Zainal Abidin et al., 2019), and in the adult hippocampus, where Drosha functions as a molecular barrier, which, by preventing oligodendrogliogenesis, restricts the multi-lineage potential of aNSCs (Rolando et al., 2016).

#### Effects in Pathological Conditions

The discovery of astrocyte-like NSCs in the hippocampus has also shed new light on the role of sncRNAs in reactive gliosis, which is increasingly viewed as an important aspect to address in order to better understand the pathological and/or age-dependent loss of neurogenesis, as well as to explore the potential of RNA therapeutics in various brain diseases. Indeed, altered expression of several miRNAs, particularly miR-146a, miR-155, and miR-132 has been reported in reactive astrocytes in the postnatal hippocampus of various neurodegenerative and ischemia models, or following status epilepticus (Aronica et al., 2010; Arena et al., 2017; Korotkov et al., 2020). Moreover, manipulation of specific miRNAs, such as miR-181a or miR-302/367, in reactive hippocampal astrocytes could ameliorate some of the neural deficits in models of focal ischemia or AD (Ghasemi-Kasman et al., 2018; Griffiths et al., 2019).

### Regulation of Adult Oligodendrogliogenesis

#### Effects in Physiological Conditions

Most knowledge on the regulation of oligodendrogliogenesis by miRNAs originates from research in embryonic development. During the embryonic development of the CNS, inhibition of miRNA formation by conditional Dicer knockout in the spinal cord resulted in a disruption of both astrogliogenesis and oligodendrogliogenesis, without affecting neurogenesis, indicating that miRNAs are essential for the initiation of gliogenesis (Zheng et al., 2010).

Similarly, the importance of miRNAs in oligodendrogliogenesis has been unveiled using Dicer ablation in OPCs. Dicer mutant mice display a lack of differentiated OPCs resulting in demyelination defects, and OPCs isolated from these mice are unable to properly differentiate *in vitro* (Dugas et al., 2010). When comparing the expression of miRNAs in OPCs and mature oligodendrocytes, a 10 to 100-fold increase of miR-219, miR-338, and miR-138 was observed. The most strongly induced miRNA among these, miR-219, directly targets genes involved in maintaining OPC proliferation, and increasing miR-219 levels stimulate OPCs to exit proliferation and start differentiation (Dugas et al., 2010; Li et al., 2019). Furthermore, transplantation of OPCs overexpressing miR-219 into a cuprizone-induced demyelinated mouse model promoted remyelination and improved cognitive function, highlighting the therapeutic relevance of miR-129-mediated regulation of oligodendrogliogenesis (Fan et al., 2017). Of interest, postnatal oligodendrocytic cell lines possess a characteristic miRNA signature composed of 43 miRNAs dynamically expressed during oligodendrogliogenesis. Within this group of 43 miRNAs, miR-9 seems of particular importance for the physiological regulation of oligodendrogliogenesis, as its expression inversely correlates with the expression of its target, the peripheral myelin protein PMP22 (Lau et al., 2008). Additionally, this study demonstrated that particular miRNAs are expressed at specific stages during OPC differentiation to mature oligodendrocytes (Lau et al., 2008). Interestingly, oligodendrogliogenesis provides a physio/pathologically relevant system for the study of functional synergy between multiple miRNAs. MiR-219 cooperates with miR-338 in regulating myelination in the CNS (Zhao et al., 2010; Wang et al., 2017a). Moreover, the increased levels of mature oligodendrocytes observed in the presence of the receptor for advanced glycation end products (RAGE) antagonist, FPS-ZM1, correlated with increased expression of miR-23a, miR-219a, and miR-338, three miRNAs associated with oligodendrocytic differentiation and remyelination (Santos et al., 2020).

While under physiological conditions aNSCs mostly produce neurons and astrocytes and not oligodendrocytes (Suh et al., 2007; Jessberger et al., 2008), hippocampal aNSCs can redirect their fate to the oligodendrocyte lineage upon genetic manipulation. The first example was provided by overexpression of the transcription factors Ascl1, Olig2, or Sox10 *in vivo*, which resulted in a switch toward the oligodendrocytic lineage (Jessberger et al., 2008; Braun et al., 2015). Follow-up studies have shown that Drosha deletion in hippocampal aNSCs impairs neurogenesis and instead induces oligodendrocytic commitment and production. Even though Drosha is part of the microRNA microprocessor, it can also directly destabilize mRNAs, suggesting that the observed regulation of aNSC fate towards oligodendrocytes may be independent of miRNAs and dependent on Drosha-mediated repression of Neurofibromin 1 (Rolando et al., 2016). Indeed, Neurofibromin 1 inactivation *in vivo* revealed a latent oligodendrocytic lineage potential of hippocampal aNSCs (Sun et al., 2015). Nevertheless, recent studies have shown that neutralization of the neurotrophin nerve growth factor promotes oligodendrogliogenesis from hippocampal neurospheres by increasing miR-219a-5p levels, highlighting the role of miRNA-dependent mechanisms in this process (Brandi et al., 2021).

#### Effects in Pathological Conditions

Many of the studies addressing the role of miRNAs in OPCs have used animal models of neurological injuries, such as stroke-induced cerebral ischemia and traumatic brain injury. In these models, a positive neurological outcome has been associated with increased oligodendrogliogenesis, which contributes to remyelination (Santra et al., 2014; Xiong et al., 2015). One of the best-characterized consequences of CNS injury is the induction of inflammation. In particular, the Toll-like receptor (TLR) proinflammatory signaling pathway regulates tissue injury (Symons et al., 2006). MiR-146(a) is an inflammation-associated miRNA that downregulates proinflammatory cytokines and TLR-mediated pathways (Taganov et al., 2006; Nahid et al., 2011). In OPCs, miR-146a is upregulated by thymosin β4, resulting in the suppression of TLR-mediated proinflammatory pathways (Santra et al., 2014). Further studies have demonstrated that thymosin β4 upregulated miR-200a in NPCs in the peri-infarct area in a model of middle cerebral artery occlusion (Santra et al., 2016). In this model, thymosin β4-mediated miR-200 upregulation resulted in increased expression of the oligodendrocyte marker myelin basic protein (MBP; Santra et al., 2016). In agreement with these studies, overexpression of miR-146a in NPCs differentiated them into OPCs, and increased the expression of myelin proteins in OPCs, indicating that miRNAs mediate some of the effects of stroke on oligodendrogliogenesis (Liu et al., 2017). Similar results were obtained in a mouse model of multiple sclerosis, where miR-146a promoted OPC differentiation and enhanced remyelination (Zhang et al., 2019a), further highlighting the involvement of sncRNAs in myelination-related disorders.

### Regulation of the Crosstalk Between NSCs and Microglia

DG microglia display distinct gene expression profiles compared to microglia found in other hippocampal regions (St-Pierre et al., 2020), suggesting that they may be specialized for crosstalk with niche cells, like aNSCs (Kreisel et al., 2019). In fact, aNSCs express a multitude of receptors for immune molecules and cytokines, allowing their regulation by microglial immune mediators (Ekdahl et al., 2009). As such, microglia may be involved in different phases of neurogenesis (Ekdahl et al., 2009), through different functions, such as initiating the inflammatory response and phagocytosis (Sierra et al., 2010, 2019; Paolicelli et al., 2011; Abiega et al., 2016).

Regulation of inflammation is one of the best characterized microglial functions and, thus, inflammatory mediators have also been studied in the context of the microglia-aNSC crosstalk (Ekdahl et al., 2003; Monje et al., 2003). However, the outcome *in vivo* is dependent on the balance of pro- and anti-inflammatory mediators in the neurogenic niche (Monje et al., 2003; Butovsky et al., 2006; Woodbury et al., 2015; Diaz-Aparicio et al., 2020). The role of microglial miRNAs in neuroinflammatory modulation has been widely documented. MiRNAs regulate the expression of many cytokines and the function of several pro-inflammatory signaling pathways in microglia, as previously reviewed (Wang and Wang, 2018; Guo et al., 2019). Microglial miRNAs such as miR-206, miR-155, and miR-32-5p, favor a pro-inflammatory microglial phenotype (Butovsky et al., 2015; Xing et al., 2016; Yan et al., 2018), while others, like miR-93, miR-367, miR-26a, miR-124, miR-199b, and miR-27a, inhibit pro-inflammatory signaling (Kumar et al., 2015; Wang et al., 2015; Louw et al., 2016; Zhou et al., 2016; Lv et al., 2017; Tian et al., 2017; Guo et al., 2019). Many of these miRNAs have an impact on the expression of cytokines that have been directly linked to aNSC fate regulation, thus, potentially regulating important aNSC features, such as proliferation and cell fate. However, there is an overall scarcity of reports directly assessing the impact of the microglial inflammation-related miRNAs on the regulation of aNSCs. One of the few studies addressing this, showed that knocking out microglial miR-155 promotes neurogenesis in NSCs cultured with LPS-activated microglia (Woodbury et al., 2015). Taken together, these observations suggest that microglial inflammation-related miRNAs may play a key role in the modulation of aNSC fate.

Phagocytosis is another of the main microglial functions that has a direct impact on aNSCs (Diaz-Aparicio et al., 2020). Microglial miRNAs also regulate microglial phagocytosis, thereby potentially altering aNSC fate. MiR-146a is mainly overexpressed by microglia in prion disease mouse brain (Saba et al., 2012). Microglial miR-146a increases by TLR2 stimulation *in vitro*, disrupting inflammatory signaling pathways and predictably targeting two phagocytic mediators of the oxidative burst (Saba et al., 2012; Guo et al., 2019). Upregulation of microglial miRNA-34a expression has been linked to decreased expression of triggering receptor expressed in myeloid/microglial cells-2 (TREM2) in microglia *in vitro* (Alexandrov et al., 2013), which has been linked to a decrease in microglial phagocytosis of Aβ42 *in vitro* (Bhattacharjee et al., 2016). Induced overexpression of miR-124, a miRNA shown to inhibit neuroinflammation and to drive macrophage differentiation towards microglia (Ponomarev et al., 2011), has also been shown to inhibit the phagocytosis of apoptotic cells in the optic tectum of zebrafish larvae (Svahn et al., 2016). Although there are no studies linking miRNA regulation of microglial phagocytosis to aNSC fate, the aforementioned miRNA-driven changes in phagocytosis could potentially regulate aNSC function and fate through changes in the microglial phagocytic secretome, as previously described (Diaz-Aparicio et al., 2020). Overall, the extent, complexity, and implications of the miRNA-driven crosstalk between microglia and aNSCs remain to be fully elucidated.

## Piwi-Interacting RNAs and Other Classes of sncRNAs

Mounting evidence has shown that some of the PIWI proteins and Piwi-interacting RNAs (piRNAs; Figure 2) are expressed in the CNS of different animal models, and participate in the processes of neurodevelopment, neurotransmission, transient focal ischemia and neurodegeneration (Kaur et al., 2018; Kim, 2019; Huang and Wong, 2021). In particular, PIWIL1 (MIWI in the mouse) and piRNAs have been linked to neuronal injury and axonal regeneration in rodents (Phay et al., 2018; Sohn et al., 2019) and *C. elegans* (Kim et al., 2018), but the underlying mechanisms are still obscure. In the mouse developing cerebral cortex, the knockdown of MIWI resulted in a multipolar morphology of neurons and a larger number of primary neurites, suggesting that the piRNA pathway can regulate neuronal migration and maturation during development (Zhao et al., 2016). The expression of PIWIL2 (MILI in mouse) protein has also been reported in the adult mouse brain, with hypomethylation of transposons and behavioral defects observed in MILI knockout mice (Nandi et al., 2016). A more recent study has described behavioral impairment in mice upon hippocampal MILI and/or MIWI depletion. Knockdown of MILI results in hyperactivity, whereas knockdown of both MILI and MIWI leads to enhanced contextual fear memory without affecting anxiety (Leighton et al., 2019).

The aforementioned studies and the majority of those which investigated the possible roles of the piRNA pathway in mammalian CNS, have focused on neurons (Dharap et al., 2011; Lee et al., 2011; Ghoshes et al., 2016; Nandi et al., 2016; Zhao et al., 2016; Leighton et al., 2019). However, as piRNA levels in these cells are low compared to germline cells, the proposed function of the piRNA pathway in synaptic plasticity and memory control (if any) is debated.

Interestingly, the hippocampus shows the highest number of unique piRNA sequences and piRNA transcripts (5494 piRNAs) in the adult brain (Perera et al., 2019). In agreement with this observation, it was recently demonstrated that the piRNA pathway has a crucial role in maintaining the homeostasis and the fate of hippocampal NSCs (Gasperini et al., 2021). In particular, the expression of MILI and piRNAs was found to be enriched in NPCs of the postnatal mouse hippocampus, compared to their differentiated progeny, and piRNA functions were shown to be essential for neurogenesis. Moreover, inhibition of the piRNA pathway in adult NPCs leads to senescence-associated inflammation and an increase in reactive astrogliogenesis (Gasperini et al., 2021), a condition which has been linked to age-related impairment of neurogenesis and neurodegeneration.

Despite the growing interest in brain-related piRNAs, the unavailability of proper methods and tools to investigate the piRNA pathway outside gonads, and therefore the lack of a fully characterized pathway in neuronal tissues, complicates research in this field. Indeed, most of the existing studies did not provide conclusive evidence that piRNA-dependent, rather than piRNA-independent, functions of these proteins drove the various phenotypes. Functions of PIWI proteins were shown to regulate chromatin structure/stability, histone/DNA methylation, DNA repair transcription, and RNA turnover and translation in gonads (Czech et al., 2018; Ozata et al., 2019), but these mechanisms are poorly investigated in CNS. Because PIWI protein functions have been attributed roles in several CNS diseases, including neurodevelopmental and neurodegenerative diseases and various cancers, a better understanding of these processes in aNSCs entails important translational implications.

With respect to additional classes of sncRNAs, at present very little is known about their involvement in the regulation of aNSC homeostasis, activation, and fate determination. Interestingly, a double-stranded RNA (dsRNA) molecule (Figure 2) expressed at the SGZ of DG, was shown to regulate fate determination of hippocampal aNSCs *in vitro* (Kuwabara et al., 2004). This dsRNA bares a recognition motif for NRSF/REST, a major negative transcriptional regulator of neuronal differentiation. Interaction between the dsRNA and the NRSF/REST machinery results in the transition from aNSCs to a neuronal (but not glial) fate, while the effects seem to be mediated by a dsRNA/protein interplay (Figure 3).

## Discussion

The complexity of the biological processes involved in aNSC homeostasis, including activation, self-renewal, and fate determination, requires tight control of numerous multilayered regulatory programs. SncRNAs, as other ncRNA species, regulate gene expression at the transcriptional and post-transcriptional level and are additionally considered to be the main players in epigenetic regulation.

In this review, we have summarized evidence indicating that sncRNAs, and primarily microRNAs, play major regulatory roles at virtually all stages of aNSC homeostasis in both health and disease. We have discussed in detail the role of individual miRNAs, in the regulation of key targets crucial for aNSC quiescence, and for their activation, which can eventually result in neurogenesis, astrogliogenesis or oligodendrogliogenesis. Moreover, we surveyed miRNA roles in the functional crosstalk between aNSCs and other relevant cell types in the adult hippocampal neurogenic niche, in particular microglia. However, with the advent of novel technologies, we have only recently begun to unveil previously hidden layers of the biology of sncRNAs. For instance, tRNA-derived fragments are a new class of sncRNAs with noncanonical biological functions unrelated to protein synthesis (Torres et al., 2019); they are produced from tRNAs by specific cleavage events catalyzed by ribonucleases, such as Dicer; and have been shown to increase during disease, where they seem to regulate stress responses, proteostasis, and neuronal survival (Fagan et al., 2021). Interestingly, tRNA-derived piRNAs, putatively requiring PIWI protein function in somatic cells, have also been identified (Keam et al., 2014). How these and other newly identified sncRNAs may independently or in synergy (with miRNAs, piRNAs, or other ncRNA species) impact phenomena related to aNSCs remains elusive.

Further contributing to our understanding of the complexity of sncRNA-mediated regulation, the functional crosstalk across and within different classes of sncRNAs has been shown to enable precise regulation of several brain functions (Mehta et al., 2021). Functional pleiotropy (each miRNA may simultaneously regulate multiple targets) and convergence onto shared targets within distinct pathways (multiple miRNAs may simultaneously regulate the same target) are two key elements of miRNA biology (Barca-Mayo and De Pietri Tonelli, 2014; Walgrave et al., 2021b) with functional relevance in AHN (for a review, see Stappert et al., 2018). Systematic interrogation of the intricate targetomes of single miRNAs or of synergistic miRNA networks in the context of aNSC regulation will enable a more accurate profiling of their regulatory repertoire and, hence, of their potential therapeutic use as mediators of regeneration of distinct cell types in the adult brain. Even though these interactions may also be critical for maintaining the heterogeneity of the adult hippocampal niche by determining the balance between neurogenesis and astrogliogenesis (Pons-Espinal et al., 2017), the majority of such mechanistic aspects have not been experimentally addressed in the context of AHN, apart from a few exceptions (Schouten et al., 2015; Jin et al., 2016; Pons-Espinal et al., 2017; Wang et al., 2017b; Bielefeld et al., 2019; Pan et al., 2019).

As an additional layer of intercellular communication, aNSCs and other cell types in the niche are capable of releasing and taking up extracellular vesicles like many other cell types in the mammalian brain (Lopez-Verrilli and Court, 2013; Luarte et al., 2016; Vogel et al., 2018). Extracellular vesicles can be divided into several categories depending on size, among which exosomes have been mostly implicated in the regulation of the aNSC niche. Exosomal content varies greatly based on the cell type from which they are released and the (patho)physiological state of the cell. In general, exosomes contain a plethora of non-coding RNAs, including miRNAs, piRNAs, tRNAs, long non-coding RNAs (lncRNAs), as well as mRNAs, lipids, and transcription factors (Valadi et al., 2007; O’Brien et al., 2020). Exosomes in the neurogenic niche have been implicated in all stages of neurogenesis, ranging from the regulation of aNSC activation (Cheng et al., 2021) to synaptic pruning of mature neuronal spines through microglia (Bahrini et al., 2015). An example of exosome-mediated intercellular signaling and synergy between multiple miRNAs is the regulation of neuroplasticity and functional recovery after stroke by the miR-17-92 cluster. Exosomes extracted from multipotent mesenchymal stromal cells and artificially enriched in miRNAs from the miR-17-92 cluster, increased oligodendrogliogenesis, neurogenesis, neurite remodeling, and neuronal dendrite plasticity in the ischemic boundary zone after stroke, possibly by activating the PI3K/protein kinase B/mechanistic target of rapamycin/glycogen synthase kinase 3β signaling pathway (Xin et al., 2017). In addition, intercellular communication *via* exosomal miRNAs has been shown to impact brain physiology and a diverse range of pathologies (Xia et al., 2019). More recently, endogenous sncRNAs, termed “glycoRNAs”, were found to bear glycan conjugations and to primarily localize on the cell membrane, putatively acting as signals in the intercellular communication and further contributing to intricate cellular interaction pathways (Flynn et al., 2021).

An issue confounding the current understanding of hippocampal aNSC biology is that a big part of our knowledge on the sncRNA-mediated regulation of aNSCs is derived from *in vitro* systems and from studies in embryogenesis or in the adult SVZ niche. Yet, evidence from heterotopical transplantation experiments demonstrated the significance of the niche microenvironment in fate choice heterogeneity of aNSCs (Beyer et al., 2020), suggesting that findings not derived from the adult SGZ should not be extrapolated to the adult DG without further experimental validation. In addition, given the context-specificity of several miRNA regulatory networks, extrapolating such observations to putative effects in AHN should be done with caution.

Another pivotal limitation is the vast knowledge gap with respect to whether the coding and non-coding mechanisms regulating aNSC quiescence, activation, and fate choice in the adult rodent brain are also conserved in humans. Studies in human systems *in vitro* and high-resolution cellular and molecular profiling of the adult hippocampal neurogenic niche in the human brain will shed more light on the existence of aNSCs, their putative heterogeneity, and their regulation in health and disease.

In conclusion, the intricate sncRNA landscape of mammalian cells has only recently started to be appreciated. Understanding the complex sncRNA-mediated regulatory mechanisms involved in aNSC homeostasis, including self-renewal, activation, and fate commitment will be key to decoding the regulatory determinants that can be used to leverage aNSC biology in brain diseases. Further studies focusing on the adult hippocampal neurogenic niche and considering the elaborate sncRNA functional communication both among them and with target pathways will be critical to profile the full regulatory potential of sncRNAs in this regard, as well as their future applications in the next generation of (RNA) therapies, particularly targeting age-related pathologies.

## Author Contributions

CF and ES conceived the article. AP, GT, OA, PB, CG, DD, CF, and ES discussed, wrote and corrected the manuscript. All authors contributed to the article and approved the submitted version.

## Conflict of Interest

The authors declare that the research was conducted in the absence of any commercial or financial relationships that could be construed as a potential conflict of interest.

## Publisher’s Note

All claims expressed in this article are solely those of the authors and do not necessarily represent those of their affiliated organizations, or those of the publisher, the editors and the reviewers. Any product that may be evaluated in this article, or claim that may be made by its manufacturer, is not guaranteed or endorsed by the publisher.
